# Prenatal Environmental Exposure to Persistent Organic Pollutants and Reproductive Hormone Profile and Pubertal Development in Dutch Adolescents

**DOI:** 10.3390/ijerph19159423

**Published:** 2022-08-01

**Authors:** Sietske A. Berghuis, Arend F. Bos, Henk Groen, Wilhelmina H. A. de Jong, Anneke C. Muller Kobold, Lucie Wagenmakers-Huizinga, Pieter J. J. Sauer, Gianni Bocca

**Affiliations:** 1 Division of Neonatology, Department of Pediatrics, Beatrix Children’s Hospital, University Medical Center Groningen, University of Groningen, 9713 GZ Groningen, The Netherlands; a.f.bos@umcg.nl (A.F.B.); saupie46@gmail.com (P.J.J.S.); 2 Department of Epidemiology, University Medical Center Groningen, University of Groningen, 9713 GZ Groningen, The Netherlands; h.groen01@umcg.nl; 3 Department of Laboratory Medicine, University Medical Center Groningen, University of Groningen, 9713 GZ Groningen, The Netherlands; hkniestdejong@saltro.nl (W.H.A.d.J.); a.c.muller@umcg.nl (A.C.M.K.); l.wagenmakers@umcg.nl (L.W.-H.); 4 Division of Endocrinology, Department of Pediatrics, Beatrix Children’s Hospital, University Medical Center Groningen, University of Groningen, 9713 GZ Groningen, The Netherlands; g.bocca@umcg.nl

**Keywords:** adolescence, adolescent, chemical exposure, endocrine disruptor, persistent organic pollutant, polychlorinated biphenyl, prenatal exposure, pubertal development, reproductive hormone, testosterone

## Abstract

Persistent organic pollutants (POPs), such as polychlorinated biphenyls (PCBs), may interfere with hormonal processes. Knowledge about the effects of prenatal exposure to PCBs and their hydroxylated metabolites (OH-PCBs) on pubertal development is limited. Therefore, the aim of the current study was to determine whether prenatal environmental PCB and OH-PCB exposure are associated with reproductive hormone levels and pubertal characteristics in 13- to 15-year-old children. In this Dutch observational cohort study, 194 mother–infant pairs were included (1998–2002). Maternal pregnancy serum levels of PCBs, OH-PCBs, and other POPs were measured. At follow-up (2014–2016), we measured serum or plasma levels of reproductive hormones in their children. We assessed Tanner stages and testicular volume (by clinician or standardized self-assessment), and participants completed questionnaires on pubertal onset. In total, 101 adolescents (14.4 ± 0.8 years; 53.7% of invited) participated, and 55 were boys. In boys, higher prenatal PCB levels were associated with higher testosterone levels, higher pubic hair stage, larger testicular volume, and younger age at onset of growth spurt and voice break. In girls, higher prenatal PCB levels were associated with higher stages for breast development. In conclusion, higher prenatal PCB exposure could be associated with more advanced pubertal development in 13- to 15-year-old children.

## 1. Introduction

There is growing evidence that prenatal environmental exposure to chemicals affects pubertal development [1,2]. There are many chemicals present in the environment because of their extensive use, their resistance to biological and chemical degradation, and bio-accumulation in the food chain. Exposure to these persistent organic pollutants (POPs) continues for long periods after the production and use has been banned by law. Humans are exposed to environmental chemicals via food, drinking-water, and air. POPs include polychlorinated biphenyls (PCBs), polybrominated diphenyl ethers (PBDEs), dichloroethane (DDE), pentachlorophenol (PCP), hexabromocyclododecane (HBCDD), and others.

PCBs are chemicals produced between 1929 and 1985 for application in a variety of products, including coolants in heat-transfer systems and lubricants in plastics [3]. PCBs are metabolized in the liver to hydroxy-PCBs (OH-PCBs). Both PCBs and OH-PCBs can be transferred over the placenta from the mother to the fetus [4]. Because OH-PCBs are transferred in a higher ratio to the fetus than PCBs, there is potentially greater toxicity of OH-PCBs [4]. The prenatal period is a vulnerable period because many developmental processes are initiated, and disruption of these processes may influence outcomes in later life [5]. Prenatal PCB exposure has been shown to interfere with neurological, immunological, metabolic, and endocrine development in children [6,7,8,9]. PCBs can interfere with hormonal pathways, including exerting estrogenic or anti-estrogenic effects [7,10]. Because pubertal development is a multifaceted process under control of several hormonal mechanisms, PCB exposure might interfere with pubertal development. Evidence is growing that exogenous hormone disruptors may advance or delay puberty [2,7,11].

Knowledge about the impact of prenatal environmental PCB and OH-PCB exposure on pubertal development is limited. Regarding prenatal PCB exposure, studies on reproductive hormone levels and pubertal stages at puberty have been performed in the USA [12,13,14], Taiwan [15,16], Russia [17], and Faroe Islands [18,19], showing inconsistent results. To the best of our knowledge, so far, no studies have been performed on the effects of prenatal OH-PCB exposure on pubertal development.

The aim of this study was therefore to determine whether prenatal environmental PCB and OH-PCB exposure is associated with pubertal development, both biochemically by measuring reproductive hormone levels and clinically by examining pubertal characteristics. In addition, we aimed to determine whether prenatal exposure to PBDEs, DDE, PCP, and HBCDD is associated with pubertal development.

## 2. Materials and Methods

### 2.1. Cohort and Study Design

This prospective longitudinal cohort study is part of the Development at Adolescence and Chemical Exposure (DACE) study, in which we followed up two Dutch cohorts. Between 1998 and 2000, 104 mother–infant pairs were included in the Risk of Endocrine Contaminants on Human Health (RENCO) study [4]. Between 2001 and 2002, 90 mother–infant pairs were included in the Groningen-Infant-COMPARE (Comparison of Exposure-Effect Pathways to Improve the Assessment of Human Health Risks of Complex Environmental Mixtures of Organohalogens) study, also known as the GIC study [20]. Children of both cohorts were invited for the current study. Six children were not invited: four had no available prenatal POP levels, one had been diagnosed with a congenital syndrome after inclusion in the cohort, and one had moved abroad. A reminder was sent in case of no response. The children were all singletons and born at term (37–42 weeks’ gestation) without congenital anomalies or diseases. Their mothers were of Western European origin, and had no serious illnesses or complications during pregnancy or delivery. At time of follow-up, all children were between 13 and 15 years (inclusion periods April 2014–December 2014 and October 2015–August 2016). All adolescents and their parents provided their written informed consent before participation in the follow-up program. The study was conducted according to the principles of the Declaration of Helsinki (adopted by the 18th WMA General Assembly, Helsinki, Finland, June 1964, and amended by the 64th WMA General Assembly, Fortaleza, October 2013) and in accordance with the Medical Research Involving Human Subjects Act (WMO). Both the original studies and the follow-up study were approved by the Medical Ethics Committee of the University Medical Center Groningen (2014/029).

### 2.2. Measurement of Prenatal POP Levels

Maternal blood samples were taken during the second and/or third trimester of pregnancy. Detailed descriptions of the analyses have been published previously [4,20]. Levels of PCB-153, 4-OH-PCB-107, 4-OH-PCB-146, and 4-OH-PCB-187 were measured in both cohorts. In the RENCO study, nine other PCBs (105; 118; 138; 146; 156; 170; 180; 183; 187) and three other OH-PCBs (3-OH-PCB-153; 3′-OH-PCB-138; 4′-OH-PCB-172) were measured, and the sum of the 10 PCBs and the sum of the 6 OH-PCBs were calculated. The following POPs were also measured in the GIC study: 2,2′-bis-(4 chlorophenyl)-1,1′-dichloroethane (p,p’-DDE), pentachlorophenol (PCP), five different polybrominated diphenyl ethers (BDEs), and hexabromocyclododecane (HBCDD). PCBs and OH-PCBs were numbered according to Ballschmiter et al. and Letcher et al. [21,22], respectively. PCB levels are given in ng/g lipid and OH-PCB levels in pg/g fresh weight.

### 2.3. Biochemical Analyses of Reproductive Hormones

Blood samples were taken by venipuncture after an overnight fast at 8.30 a.m., except for three samples which were taken around 10.30 a.m. One BD Vacutainer K2E (EDTA) 18.0 mg of 10 mL was used for obtaining plasma samples, and a BD Vacutainer SST II Advance of 5 mL for obtaining serum samples (Becton Dickinson, Franklin Lakes, NJ, USA). The samples were centrifuged (4 °C; 211 r (mm); 2347 n (rpm); 1300 RZB (g); t (min) = 11.20), and the plasma and serum were transferred to plastic tubes, and stored at −20 °C until analysis. Estradiol (E2; interassay CV of 3.1–6.0%; limit of detection, LOD = 0.02 nmol/L plasma) and Luteinizing Hormone (LH; interassay CV 2.6–4.2%; LOD = 0.1 U/L plasma) were analyzed by electrochemiluminescent immunoassays on the Roche Hitachi e501 analyzer (Roche, Basel, Switzerland). Follicle Stimulating Hormone (FSH; interassay CV 3.5–4.5%; LOD = 0.1 U/L plasma) was performed on the AutoDELFIA immunoassay analyzer of Perkin Elmer (Perkin Elmer, Groningen, the Netherlands). Anti-Müllerian hormone (AMH; interassay CV 8.0–9.0%; LOD = 1.5 ng/mL serum) was assayed using the AMH Gen II Enzyme Linked Immunosorbent Assay (ELISA) of Beckman Coulter (Beckman Coulter, Woerden, The Netherlands). Inhibin B (interassay CV 4.1–7.0%; LOD = 2.91 pg/mL serum) was assayed using the Inhibin B Gen II ELISA (Beckman Coulter). Both ELISAs were processed on a DS2 automated ELISA processing system (Dynex Technologies, Chantilly, VA, USA). SHBG (sex hormone-binding globulin; interassay CV 5.5–5.7%; LOD < 0.1 nmol/L plasma) was analyzed on an Architect Chemiluminescent Microparticle immunoanalyzer (Abbott Diagnostics, Hoofddorp, The Netherlands). Albumin (interassay CV 2.0–2.2%; LOD = 2 g/L serum) was analyzed by bromcresol green (BCG)-based colorimetric assay on a Roche Hitachi c501 analyzer (Roche, Basel, Switzerland). Testosterone (interassay CV 2.4–5.0%; limit of quantitation = 0.04 nmol/L plasma) was measured by an in-house-developed liquid chromatography tandem mass spectrometric method (LC-MS/MS) with online solid-phase extraction (SPE). LC was performed by an Acquity Online Sample Manager system from Waters (Milford, MA, USA) using a Kinetex C18 column (2.6 µm, 100 × 2.1 mm) from Phenomenex (Torrance, CA, USA). For online SPE, Waters XBridge™ C8-cartridges (10 × 1 mm) were used. Detection was performed by a Waters Xevo TQ-S tandem mass spectrometer in positive ionization and multiple reaction monitoring mode. Data were analyzed using TargetlynxTM software (Waters). Free testosterone was calculated using the formula of Vermeulen, using testosterone, albumin, and SHBG results obtained with the methods described above [23,24].

### 2.4. Outcome Measures on Pubertal Development

Pubertal development was staged according to Marshall and Tanner [25,26]. For both boys and girls, it included a stage for pubic hair, for boys a stage for genital development, and for girls a stage for breast development, which can all range from stage 1 to stage 5, with a higher stage reflecting a more advanced development. Pubic hair in boys can also reach stage 6, but this stage is not usually reached before the mid-twenties, and if it occurred in our study it was rated as stage 5 [26]. Tanner stages were assessed at the clinic by author SAB, trained by pediatric endocrinologist author GB, or by the participants themselves using a validated method for self-assessment of pubertal stages [27,28]. This method of Carel et al. includes realistic-colored pictures of the Tanner stages 1 to 5 [27]. In addition to the pictures, the children received instructions, looked in a mirror, and had the opportunity to ask for clarification. The children were asked to stick a note on the pictures that best reflected their current stage of pubertal characteristics. Testicular volume in mL was assessed using a Prader orchidometer, by author SAB or by the participants themselves. The volume of the largest testicle was used in the statistical analyses. A questionnaire on onset of pubertal characteristics was filled in at the clinic. At the clinic, height was determined to the nearest 0.1 cm, and weight to the nearest 0.1 kg, using the same stadiometer (Ulmer Stadiometer) and standard calibrated scale for all measurements. Measurements of height and weight were performed in duplicate, with the children only wearing their underwear. The average of both measurements was used for the calculation of the body mass index (BMI), by dividing the weight in kilograms by the height in meters squared. Parents reported maternal age at menarche and paternal timing of growth spurt. The investigators were blinded to prenatal POP levels.

### 2.5. Statistical Analyses of Data

*T*-tests were used to compare POP levels between the participating and non-participating children and between the children with and without reporting an onset of pubertal characteristics. *T*-tests were also used to compare POP levels and reproductive hormone levels between children included in the RENCO and GIC cohorts. Spearman’s rank correlation test was used to assess whether Tanner stages and reported ages at onset of pubertal characteristics correlated with each other. Pearson’s correlation test was used to assess whether testosterone level correlated with testicular volume.

First, the POP levels were log10 transformed. With regard to the categorical variable Tanner stage, dummy variables were made with Tanner stage 4 as a reference category. Multivariable regression analyses (method: Enter) were performed to assess associations between prenatal POP levels and levels of reproductive hormones, testicular volume, Tanner stages, and self-reported ages at onset of pubertal characteristics.

We considered the following factors as confounders: (1) for the analyses on reproductive hormone levels and testicular volume: the age at examination (in months) and BMI; (2) for the analyses on Tanner stages: the age at examination (in months), BMI, maternal age at menarche (only for girls), timing of growth spurt of the father (only for boys; early versus average/late compared to peers), and assessor of Tanner stages (SAB versus participant); (3) for the analyses on self-reported ages at onset of pubertal characteristics: the BMI, maternal age at menarche (only for girls), and the timing of growth spurt of the father (only for boys; early versus average/late compared to peers). A factor was considered as a confounder and included in the multivariable linear regression model if it had a *p*-value < 0.05 in univariable analyses. With regard to the Tanner stages, the confounder was included if at least one dummy variable was associated with the confounding factor in multivariable analyses with a *p*-value < 0.05. To assess whether there are ‘U’-shaped relationships between prenatal PCB and OH-PCB levels and reproductive hormone levels, we computed dummy variables for Q2 plus Q3 (versus Q1 plus Q4) and included this variable in the multivariate linear regression models. A *p*-value below 0.05 was considered statistically significant, and between 0.05 and 0.10 was considered a trend towards significance. The Statistical Package for the Social Sciences, version 23 (SPSS; IBM Corp., Armonk, NY, USA) was used.

## 3. Results

### 3.1. Study Group

Of the 188 children invited, 101 (53.7%) participated, 44 (23.4%) declined the invitation, and 43 (22.9%) did not respond. The final study group consisted of 55 boys and 46 girls. All children were 13–15 years old, except one girl who turned 16 the day prior to the follow-up visit. Almost all children, except one boy and girl, lived in the northern part of the Netherlands at the time of follow-up. Characteristics of the study group are presented in Table 1.

### 3.2. Prenatal POP Levels

The prenatal POP levels of the children participating in the current follow-up program have been reported previously [29]. The median PCB-153 level was 76.7 ng/g lipid (IQR: 52.0–104.6), the median Σ 10 PCBs was 319.0 ng/g lipid (IQR: 244.2–401.1), and the median Σ 6 OH-PCBs was 377.5 pg/g fresh weight (IQR: 276.5–540.5). Boys included in the RENCO cohort had significantly higher mean prenatal levels of PCB-153, OH-PCB-107, and OH-PCB-187 compared with boys included in the GIC cohort (Table 2). Girls included in the RENCO cohort had significantly higher mean prenatal levels of PCB-153, OH-PCB-107, and OH-PCB-187, and significantly lower OH-PCB-146 compared with girls included in the GIC cohort (Table 2). The POP levels did not differ between the in- and excluded children, except for PBDE-154, which was lower in included children (0.497 ± 0.241 versus 0.837 ± 0.733 ng/g lipid; t = −2.573; *p* = 0.028).

### 3.3. Levels of Reproductive Hormones

In Table 3, we present the median levels of reproductive hormones and albumin in plasma or serum samples of the adolescents. Boys included in the RENCO cohort had significantly higher levels of testosterone, free testosterone, E2, LH and FSH, and significantly lower SHBG, compared to boys included in the GIC cohort (Table S1). There was no significance difference in the mean reproductive hormone levels measured in girls included in the RENCO cohort compared with girls included in the GIC cohort (data not shown). Nine girls used contraceptives and were excluded for the analyses on the associations between POP levels and reproductive hormone levels. For nine children, no plasma samples were available due to logistic reasons. For samples with reproductive hormone levels below the LOD, the level was taken as LOD divided by two.

### 3.4. Pubertal Characteristics

Outcomes on pubertal development are shown in Table 1. For two boys, Tanner stages were not written down after self-assessment, and for one, the value of self-assessed testicular volume (value: 3 mL) was excluded from analyses due to discrepancy with self-assessed Tanner stages and onset of pubertal characteristics outcomes (all reflecting more advanced pubertal development: Tanner stage 3, and 12 years at first ejaculation, onset pubic hair growth, and onset growth spurt, and 14 years at voice break). Tanner stages correlated strongly with each other, and Tanner genital and pubic hair stages correlated with testicular volume and testosterone levels (Table 4a). Tanner stages and testicular volume did not correlate with self-reported onset of pubertal characteristics in our study. Higher testosterone levels correlated with larger testicular volume (Table 4a; Pearson’s *r* = 0.45; *p* = 0.002), irrespective of whether the volume was assessed by the clinician (rho = 0.711; *p* = 0.004; *n* = 14), or by the child using self-assessment (rho = 0.385; *p* = 0.027; *n* = 33). Regarding girls, two refused pubertal assessment, and for two girls, Tanner stages were not written down after self-assessment. For one girl, assessment of pubic hair was not possible due to shaving. Tanner breast stage correlated with Tanner pubic hair stage in girls (Table 4b). Pubic hair stage correlated negatively with ages at onset of menarche, breast growth, and growth of pubic hair, and showed a negative trend with age at growth spurt (Table 4b). Breast stage did not correlate with self-reported ages at onset of pubertal characteristics.

### 3.5. Confounding Factors

In boys, a higher BMI was associated with higher free testosterone, E2, and LH, and with lower SHBG, larger testicular volume, and younger age at onset growth spurt (Table 5). Boys with fathers reporting an average/late onset of growth spurt had a higher age at onset of growth spurt compared with boys with fathers who reported an early onset of growth spurt (beta = 0.432; *p* = 0.015). In girls, BMI was not significantly associated with levels of reproductive hormones. A higher age of maternal menarche was associated with higher ages at onset of breast growth (beta = 0.46; *p* = 0.00) and menarche (beta = 0.37; *p* = 0.03). For girls, Tanner pubic hair stage 3 was associated with higher age of maternal menarche (beta = 0.33; *p* = 0.05), using Tanner stage 4 as a reference category.

### 3.6. PCBs and Pubertal Development in Boys

**Figure 1:**
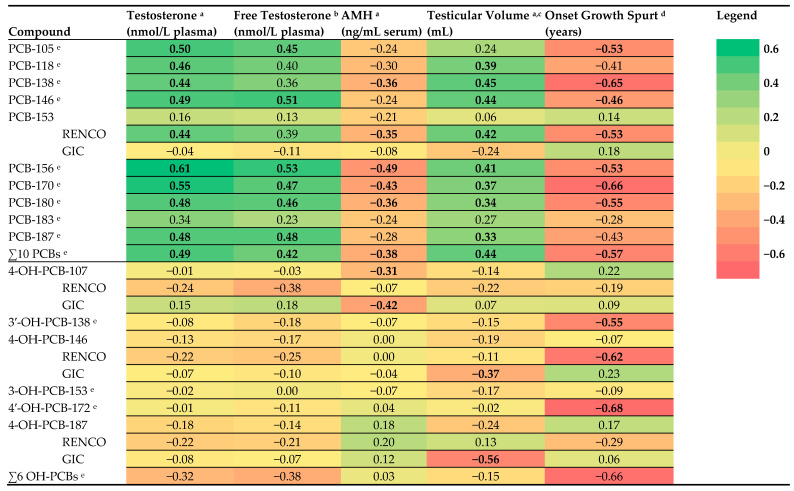
Heat map of adjusted beta coefficients obtained from linear regression analyses performed between log10 transformed prenatal levels of polychlorinated biphenyls (PCBs) and hydroxylated PCBs (OH-PCBs) and reproductive hormone levels and pubertal characteristics in boys. AMH: anti-müllerian hormone; associations with a *p*-value < 0.10 are shown in bold; ^a^ adjusted for age at examination; ^b^ adjusted for age and body mass index (BMI); ^c^ volume of the largest testicle and measured with an orchidometer; ^d^ adjusted for BMI and onset paternal growth spurt; only boys who reported ‘yes’ on the question whether they mentioned onset of growth spurt were included; RENCO cohort: Risk of Endocrine Contaminants on Human Health cohort; GIC cohort: Groningen-Infant-COMPARE (Comparison of Exposure-Effect Pathways to Improve the Assessment of Human Health Risks of Complex Environmental Mixtures of Organohalogens) cohort; ^e^ RENCO cohort.

Mainly positive associations were found between prenatal PCB levels and pubertal development in boys, as reflected by higher plasma testosterone levels, more advanced Tanner stages, larger testicular volumes, and/or younger ages at onset of pubertal characteristics (Figure 1). Eight of the ten PCBs and the sum of all ten PCBs were positively associated with testosterone, and five PCBs with free testosterone (Table S2). With regard to AMH levels, higher prenatal levels of two PCBs were found to be associated with lower AMH levels at adolescence, and similar trends were found for three other PCBs and the sum of all ten PCBs (Table S2; Figure 1). In boys included in the GIC cohort, PCB-153 was not significantly associated with reproductive hormone levels (Figure 1 and Table S2). With regard to testicular volume, higher prenatal levels of five PCBs and the sum of all ten PCBs were associated with a larger testicular volume, and similar trends were found for three other PCBs (Table S3). With regard to Tanner stages, comparing lower stages for pubic hair (stages 2 and 3) with stage 4 showed that lower stages were negatively associated with prenatal levels of PCBs (Table S4). With regard to genital stages, stage 3 was negatively associated with prenatal PCBs compared with stage 4 (Table S4). Higher prenatal levels of PCBs were found to be associated with a younger age at onset of growth spurt and a younger age at voice break in the boys who reported that they noticed the onset of the specific pubertal characteristic (Table S3). PCB-105 and PCB-118 levels were higher in boys reporting first ejaculation than in boys who did not (7.30 versus 3.14 ng/g lipid; t = −2.391; *p* = 0.027 and 27.91 versus 14.78 ng/g lipid; t = −3.298; *p* = 0.003, respectively).

### 3.7. PCBs and Pubertal Development in Girls

**Figure 2:**
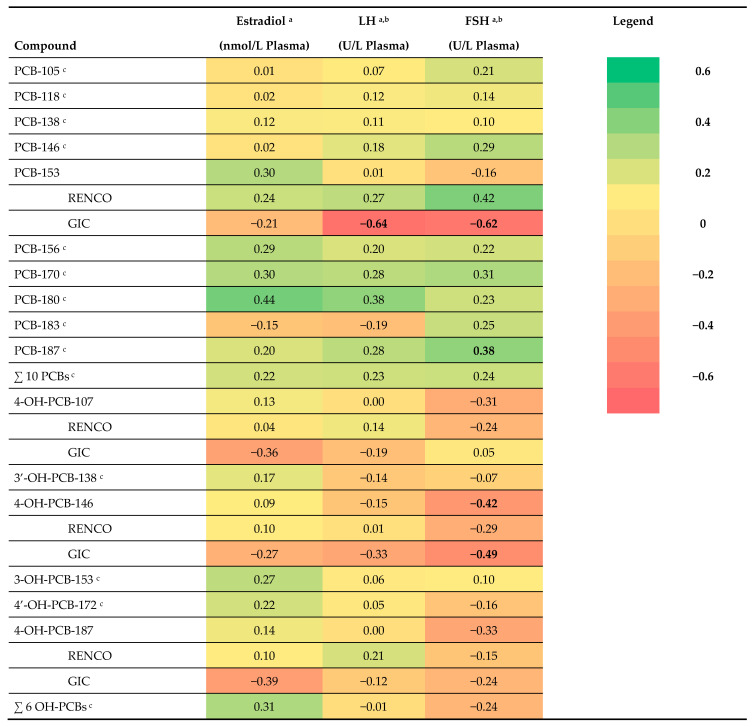
Heat map of adjusted beta coefficients obtained from linear regression analyses performed between log10 transformed prenatal PCB and OH-PCB levels and reproductive hormone levels in girls. LH: luteinizing hormone; FSH: follicle stimulating hormone; associations with a *p*-value < 0.10 are shown in bold; ^a^ adjusted for age at examination in months; ^b^ 1 sample < LOD, taken as LOD/2 in analyses; ^c^ RENCO cohort.

Compared to boys, we found fewer significant associations for girls between prenatal PCB levels and pubertal development. Some positive associations were found between prenatal PCB levels and pubertal development in girls, as reflected by higher levels of reproductive hormones and more advanced pubertal stages (Tables S5 and S6). Based on visual inspection of the heat map of the adjusted beta coefficients obtained from linear regression analyses, there seems to be a pattern that higher prenatal PCB levels may be associated with higher levels of E2, LH, and FSH at adolescence (Figure 2). In contrast to the findings in the RENCO cohort, higher PCB-153 levels were negatively associated with LH and FSH in the girls included in the GIC cohort (Figure 2 and Table S4). Four PCBs were positively associated with a higher stage for breast development (stage 5 compared to stage 4), and similar trends were found for one other PCB and for the sum of all ten PCBs. Two PCBs and the sum of all ten PCBs were negatively associated with a lower breast stage (stage 3 compared to stage 4) (Table S6). No clear pattern was found between prenatal PCB levels and Tanner stage for pubic hair in girls, only PCB-153 and PCB-156 were negatively associated with stage for pubic hair (stage 3 compared to stage 4). None of the PCBs were associated with self-reported age at onset of pubertal characteristics (Table S7).

### 3.8. OH-PCBs and Other POPs and Pubertal Development

With regard to prenatal OH-PCB levels and pubertal development in boys, no specific pattern of associations was found with reproductive hormone levels and pubertal characteristics, although non-significant associations were found in seemingly opposite directions compared to the found associations for PCB levels (Figure 1; Table S2). The compound 4-OH-PCB-107 was associated with older age at first ejaculation, and the compound 4-OH-PCB-187 with a smaller testicular volume. The compound OH-PCB-146 was associated with lower FSH in girls in the total cohort (Table S5). For some OH-PCBs, ‘U’-shaped or inverted ‘U’-shaped associations were found with reproductive hormone levels, but the sample sizes were too small to draw conclusions (data not shown).

Regarding p,p′-DDE, PBDEs, PCP, and HBCDD, measured in the GIC cohort, some associations were found with reproductive hormone levels at adolescence (Tables S8–S10). Higher prenatal PCP levels were associated with lower Inhibin B (beta = −0.42; *p* = 0.02), and a smaller testicular volume (beta = −0.37; *p* = 0.05) in boys. In girls, higher prenatal PCP levels were associated with higher E2 levels at adolescence (beta = 0.71; *p* = 0.01).

## 4. Discussion

Our explorative study shows that higher prenatal exposure to PCBs was associated with outcomes indicating a more advanced pubertal development in boys, and, to a lesser extent, with more advanced pubertal development in girls. To our knowledge, this is the first study in which both the biochemical and clinical assessments indicated an advanced pubertal development after higher prenatal PCB exposure. In boys, higher prenatal exposure to PCBs was associated with higher testosterone levels, higher pubic hair stage, with larger testicular volume, and younger ages at onset of growth spurt and voice break. In girls, higher prenatal PCB levels were associated with higher stages for breast development. With regard to OH-PCBs, PBDEs, DDE, PCP, and HBCDD, no specific pattern of associations was found with pubertal development in our exploratory study.

### 4.1. Prenatal PCB Exposure and Higher Testosterone Levels in Boys

The finding that, in boys, higher prenatal exposure to PCBs was associated with higher levels of testosterone at adolescence has not been reported previously [1]. An overview of the results of studies on the associations between prenatal PCB exposure and reproductive hormone profile, Tanner stages and testicular volume can be found in Table 4 of the overview article by Berghuis et al. [1]. Only Mol et al. reported that, based on visual inspection of their data, in 156 14-year-old boys on the Faroe Islands, higher prenatal PCB levels were associated with higher testosterone levels, but it did not reach statistical significance [19]. Our findings are in contrast with some studies reporting no associations between prenatal PCB exposure and testosterone levels in boys at (peri-)pubertal age in the USA and Taiwan [13,15,30]. Possible explanations for the fact that Su et al. did not find associations with testosterone levels can be that the boys in their study were younger (8 years old) with undetectable testosterone levels in some boys, and a smaller sample size (23 boys) than in our study [30]. The finding that higher prenatal PCB exposure was associated with higher testosterone levels in boys is in line with our results on clinical assessments and questionnaires, showing a higher pubic hair stage, larger testicular volume, and younger ages at onset of growth spurt and voice break.

### 4.2. Prenatal PCB Exposure and Larger Testicular Volume and Higher Pubic Hair Stage in Boys

Our finding of advanced clinically assessed pubertal development after higher prenatal PCB exposure is in line with a study by Humblet et al. reporting that higher maternal PCB levels (8–9 years after pregnancy) were associated with earlier pubertal onset, as reflected by Tanner genital stages ≥ 2, but not with Tanner pubic hair stages ≥2, or a testicular volume > 3 mL in 489 Russian boys [17]. In contrast to our study, four studies reported no associations with pubertal development in boys [1,14,15,19,30]. A possible explanation for the fact that Su et al. did not find associations with pubertal stage and testicular volume could be that the boys in their study were younger (8 years old) compared to the boys in our study [30]. Hsu et al. used a different estimation of PCB exposure and other statistical analyses compared with our study: they did not measure prenatal PCB levels, but compared mothers with ‘Yucheng’ oil disease with an ‘unexposed’ group. The use of other statistical methods, for example, continuous values for prenatal PCB exposure, instead of a dichotomized estimated exposure measure, might be an explanation for the differences between the outcomes in the cohorts. Additionally, it might be possible that even the ‘unexposed’ group in the study by Hsu et al. had been exposed to environmental levels of PCBs. It might be interesting to compare the prenatal PCB levels in the ‘unexposed’ group with the background exposure levels measured in our cohort. Gladen et al. performed statistical analyses on the age of attainment of pubertal stage and assessment of pubertal development was performed by self-assessment at home, where-as in our study the assessment of pubertal stage was measured at the clinic by a clinician or by the participants with the opportunity to ask for clarification [14]. In the study by Mol et al. PCB levels were measured in cord tissue, compared to maternal serum in our study [19]. Inverse associations between prenatal PCB exposure and pubic hair development, and weak, non-significant inverse associations between prenatal PCB exposure and genital stage and testicular volume were found in a study in 438 boys on the Faroe Islands [18]. Although it is difficult to compare the exposure levels due to differences in assessment methods, the prenatal exposure levels in the latter study on the Faroe Islands (with the traditional habit of eating pilot whale blubber) were estimated to be much higher than the prenatal exposure levels in boys in our cohort: the estimation for twice the sum of the PCB congeners 138, 153, and 180 was 643.33 ng/g lipid cord blood (calculated based on 3 g/L lipid in cord serum; using an estimation that the cord blood levels are about 60% of maternal levels, it might suggest that maternal serum levels could be above 1000 ng/g lipid) versus 416.29 ng/g lipid in maternal serum in our study. Our findings might implicate that even relatively low prenatal PCB exposure could interfere with pubertal development. Another difference between the study by Grandjean et al. and our study is that the children in the study of Grandjean et al. were included between 1986 and 1987, whereas in our study the children were included between 1998 and 2002. Subsequently, the pubertal development was assessed about 15 years later in time in our study than in the study by Grandjean et al. Because during recent years an ongoing secular trend towards earlier pubertal timing has been observed [31], this might have also led to differences in pubertal development between the two studies. The mean age at assessment of the pubertal development was almost similar for the studies, 13.8 years in the study by Grandjean et al. compared with 14.4 years in our study. The mean testicular volume, assessed with an orchidometer, was slightly larger in our study compared with the mean volume in the study of Grandjean et al. (10 versus 9.4 mL), and the percentage of boys having Tanner pubic hair stage 4 or 5 was higher (49 versus 38%), but the percentage of boys having Tanner genital stage 4 or 5 was lower in our study (28 versus 43%). We predominantly found associations between prenatal exposure to PCBs and pubic hair stage and testicular volume, which were both relatively more advanced than the pubertal development in the study by Grandjean et al. This might also be an explanation for differences between our study and the study by Grandjean et al. regarding the findings on the effects of prenatal exposure to PCBs on pubertal development.

### 4.3. Prenatal PCB Exposure and Higher FSH in Girls

The finding, based on visual inspection of the heat map of adjusted beta coefficients, that higher prenatal PCB exposure is associated with higher FSH in girls, is in line with a study by Yang et al. [16]. They reported a weak, marginally significant, association with higher FSH levels in 20 Taiwanese adolescents born to mothers having symptoms of ‘Yucheng’ oil disease or a history of consumption of contaminated rice oil, compared to 18 unexposed controls [16]. Comparing our results with the study of Yang et al. has several limitations, for example, due to differences in statistical analyses and differences in exposure measurements. To the best of our knowledge, there are no other studies reporting on associations between prenatal PCB levels and reproductive hormone levels in girls at adolescence. In peri-pubertal Taiwanese girls at 8 years of age, no associations were found between prenatal PCB levels and FSH or LH [30], which might be explained by the fact that the levels are too low at that age to find effects. Our findings of advanced pubertal development, as reflected by higher FSH and LH levels in girls, is in line with the findings on clinically assessed pubertal development, for example, higher stages of breast development and pubic hair.

### 4.4. Prenatal PCB Exposure and Higher Breast Stage in Girls

The finding that higher prenatal exposure to PCB-153 was associated with higher breast stage has not been reported previously. Two American studies reported no associations between prenatal exposure to PCBs and breast or pubic hair stage [12,14] (for review see Mouritsen et al. [32]). A possible explanation for why we did find associations whereas others did not might be differences in test method: both studies only used self-assessment at home, which might be less precise than assessment by a clinician or by self-assessment at the clinic after instructions with the opportunity to ask for clarification. Both American studies used only total PCBs, whereas we investigated also individual PCBs. The maternal levels of PCB-153 measured in the cohort, followed up by Gladen et al., were comparable to levels in our study group (80 versus 77 ng/g lipid), whereas PCB-153 levels were higher in Michigan studies [14,33]. Since PCB-153 is the most abundant PCB in humans, confirmed by our study [34], a possible explanation might be that only this PCB congener was found to be associated with pubic hair stage in girls. A possible explanation for the higher pubic hair stage might be an increase in the production of adrenal androgens, because they are responsible for growth of pubic hair in girls. A study in a human in vitro model showed that several chemicals can disturb adrenal steroidogenesis, but effects of PCBs were not assessed in that study [35]. Whether PCB-153 might influence pubic hair development in girls by disturbing adrenal androgen levels during puberty has not been studied yet.

### 4.5. Prenatal OH-PCB Exposure and Pubertal Development

In contrast to the reported positive associations between prenatal PCB exposure and pubertal development, visual inspection of the data on OH-PCBs suggests that prenatal OH-PCB exposure might be non-significantly associated with lower levels of several reproductive hormones. To the best of our knowledge, there are no other studies reporting on the effects of prenatal OH-PCB exposure on reproductive hormones levels; therefore, there are no studies to compare our results with.

### 4.6. Different Findings Cohorts

The reported patterns of significant associations between prenatal PCB exposure and pubertal development were mainly found in the children included in the RENCO cohort and born between 1998 and 2000. In the combined cohort, prenatal PCB-153 levels were lower in children with less advanced pubic hair stages, in boys and in girls, which was consistent for both cohorts separately. In contrast, PCB-153, the only PCB compound measured in the GIC cohort between 2001 and 2002, was not significantly or marginally significantly associated with reproductive hormone levels in boys and girls. Possible explanations for these differences in findings between the cohorts might be that the maternal levels of PCB-153 measured from 2001–2002 in the GIC cohort were lower compared to levels measured from 1998–2000 in the RENCO cohort, and/or that the children included in the RENCO cohort were included at an older age with a more advanced pubertal development compared to children included in the GIC cohort. Larger longitudinal cohort studies on the link between prenatal PCB exposure and pubertal development are needed to answer the question of whether current environmental PCB exposure levels are associated with more advance pubertal development, as was found for children born from 1998–2000.

### 4.7. Possible Mechanism

There are not many studies reporting on underlying mechanisms for an up-regulation of reproductive hormone levels and larger testicular volumes after higher prenatal PCB exposure. One possible underlying mechanism could be a decrease in expression of DNA methyltransferases, as has been found in mice exposed to PCBs [36]. With regard to a study in mice, He et al. reported that intra-uterine exposure to PCB-118 was associated with an increased testicular coefficient (expressed as percentage of organ weight in live body weight) in male offspring compared to unexposed mice. They reported histological changes in the testes after intra-uterine PCB-118 exposure, including larger diameter of the seminiferous tubules, larger height of the seminiferous epithelium, and a smaller relative number of spermatogonia, compared to unexposed mice. He et al. also looked into mRNA expression, and found that intra-uterine exposure to PCB118 decreased the relative expression levels of DNA methyltransferases and its co-regulatory factor Uhrf1 in the testes of offspring in a dose-dependent manner. This study in mice suggested that intra-uterine exposure to a PCB congener can be positively associated with testicular volume in offspring, but also with histological changes and changes in mRNA. Further studies are needed to explore whether higher prenatal environmental PCB exposure is associated with sperm quality.

### 4.8. Strengths and Limitations

A strength of our study is that we assessed pubertal development both biochemically and clinically. This gave us the opportunity to compare the findings on both assessments. A second strength is that almost all children were still living in the northern part of the Netherlands, which minimizes the variability in postnatal exposure levels due to the living area. A third strength is that the time between onset of pubertal characteristics and recall was relatively short, because we included children at ages between 13 and 15 years. A fourth strength is that the pubertal questionnaires were filled in at the clinic, giving the adolescents the opportunity to ask for clarification, and thus minimizing the number of lacking or unclear answers. A final strength is that we were able to adjust for a reliable BMI, because height and weight were measured at the clinic.

We noticed several limitations. Firstly, there was a possibility of Type 1 errors due to the large number of comparisons, which might result in chance findings. However, we found significant associations between prenatal PCB and biochemical measurements as well as with clinically assessed pubertal development, which is coherent, which we believe is rather unlikely to be explained only by chance. We believe that our analyses were justified as part of a careful evaluation of a rich data set in hypothesis-driven research [37]. Secondly, there was a possibility for bias due to the way of recruitment of the pregnant women. The women who were willing to participate in a study on the effects of chemical exposure might be more aware of their lifestyle and eating habits, and possibly adapt their lifestyle, which might have lowered their POP exposure. As a consequence, the general Dutch population might have even higher exposure levels compared to our study group, which may have an even greater impact on pubertal development.

Whether our findings of advanced pubertal development have consequences for later life needs to be studied. Associations were reported between pubertal development and cancer risk during later life. In girls, earlier onset of pubertal development was found to be related to breast cancer [38,39]. In boys, older age at sexual maturation (using single nucleotide polymorphisms associated with Tanner genital stage as surrogate for pubertal changes) was found to be related to a reduced risk of later prostate cancer [40]. A study performed in Sweden showed that mothers of men with testicular cancer had higher serum levels of PCBs compared to control mothers, whereas there was no statistically significant difference in serum PCB levels of the cases and controls [41]. Whether children with higher prenatal environmental exposure also have higher POP levels during adolescence needs to be investigated, as also whether the impact of POPs on pubertal development is mainly due to prenatal or postnatal exposure.

## 5. Conclusions

Higher prenatal environmental PCB exposure could be associated with more advanced pubertal development, based on biochemical and clinical parameters, in 13- to 15-year-old Dutch children. These patterns of associations were mainly observed in the sub-cohort of 14- to 15-year-old children born between 1998 and 2000, and not in the sub-cohort of 13- to 14-year-old children born between 2001 and 2002. In boys, higher PCB exposure was associated with higher testosterone levels, more advanced pubic hair stage, larger testicular volume, and younger age at onset growth spurt and at voice break. In girls, higher prenatal PCB exposure was found to be associated with more advanced stage for breast development. No specific pattern of associations was found between prenatal OH-PCBs, PBDEs, DDE, PCP, and HBCDD, and pubertal development. Our findings raise concern towards the effects of prenatal exposure to man-made compounds on pubertal development.

## Figures and Tables

**Table 1:** Characteristics of the study group (N = 101).

Characteristic	Value				
Sex, boy/girl	55/46 (54.5/45.5%)				
Gestational age at birth (weeks)	40 (37–42)				
Age at examination (years) -Total cohort	14.4 ± 0.8				
-RENCO cohort (*n* = 54)	15.1 ± 0.4				
-GIC cohort (*n* = 47)	13.6 ± 0.3				
Body mass index at examination	20.0 ± 3.6				
Assessment Tanner stage by clinician [yes/no] (*n* = 97)	33/64 (34/66%)				
Maternal education level					
Below average (≤11 years education)	9				
Average (12–13 years education)	41				
Above average (≥14 years education)	51				
Maternal smoking during pregnancy [yes/no]	13/88 (13/87%)				
Maternal alcohol consumption during pregnancy [yes/no]	21/80 (21/79%)				
Maternal age at menarche (*n* = 97; years) Paternal timing growth spurt (*n* = 75) [early–average/late]	12.8 ± 1.5 47/28 (63/37%)				
**Assessment of pubertal stage**					
Boys: -Testicular volume (*n* = 54; mL)	10.0 ± 3.8				
**Stage**	**1**	**2**	**3**	**4**	**5**
-Tanner genital stage (*n* = 53)	2	14	22	14	1
-Tanner pubic hair stage (*n* = 53)	4	8	15	23	3
Girls: -Tanner breast stage (*n* = 42)	0	0	15	17	10
-Tanner pubic hair stage (*n* = 41)	0	0	17	21	3
**Questionnaire on pubertal development**					
Boys: -‘Did you observe growth of pubic hair?’ [yes] -‘If yes: from which age?’ (years)	49 (89%)		12.4 ± 0.92		
-‘Did you notice that you have grown a lot in short time?(growth spurt)’ [yes] -‘If yes: from which age?’ (years)	44 (80%)		12.4 ± 1.10		
-‘Have you had ejaculations?’ [yes] -‘If yes: from which age?’ (years)	29 (53%)		13.0 ± 0.69		
-‘Did you notice voice break (lower voice)’ [yes] -‘If yes: from which age?’ (years)	35 (64%)		13.2 ± 0.76		
Girls: -‘Did you observe breast growth?’ [yes] -‘If yes: from which age?’ (years)	46 (100%)		11.7 ± 1.20		
-‘Did you notice that you have grown a lot in short time?(growth spurt)’ [yes] -‘If yes: from which age?’ (years)	37 (80%)		11.7 ± 1.37		
-‘Did you observe growth of pubic hair?’ [yes] -‘If yes: from which age?’ (years)	46 (100%)		12.0 ± 1.06		
-‘Have you had a period?’ [yes] -‘If yes: from which age?’ (years)	39 (85%)		12.4 ± 1.16		

Data are given as frequencies (*n*/*n*), median (min-max), or mean ± SD; RENCO cohort: Risk of Endocrine Contaminants on Human Health cohort; GIC cohort: Groningen-Infant-COMPARE (Comparison of Exposure-Effect Pathways to Improve the Assessment of Human Health Risks of Complex Environmental Mixtures of Organohalogens) cohort.

**Table 2:** *T*-tests comparing means of prenatal levels of PCB-153 and OH-PCBs between the RENCO and COMPARE cohorts for children included in the DACE study.

		Boys						Girls					
Compound	Cohort	*n*	Mean	SD	SEM	t	*p*-Value	*n*	Mean	SD	SEM	t	*p*-Value
PCB-153 ^a^	RENCO	26	93.19	41.00	8.04	2.77	0.008	28	110.53	40.11	7.58	4.67	<0.001
	COMPARE	29	63.90	37.35	6.94			18	65.77	24.84	5.85		
OH-PCB-107 ^b^	RENCO	26	74.73	34.72	6.81	6.31	<0.001	26	83.08	60.04	11.78	3.98	<0.001
	COMPARE	28	27.59	16.29	3.08			17	31.94	21.29	5.16		
OH-PCB-146 ^b^	RENCO	26	82.42	34.71	6.81	−1.22	0.227	26	84.85	42.75	8.38	−2.57	0.016
	COMPARE	29	95.23	42.03	7.81			18	130.69	66.81	15.75		
OH-PCB-187 ^b^	RENCO	26	137.38	44.58	8.74	6.54	<0.001	26	149.85	58.36	11.44	3.37	0.002
	COMPARE	29	73.94	22.64	4.20			18	96.95	38.17	9.00		

RENCO cohort included 1998–2000; GIC cohort included 2001–2002; DACE study: Development at Adolescence and Chemical Exposure study; ^a^ in ng/g lipid; ^b^ in pg/g fresh weight.

**Table 3:** Levels of reproductive hormones in plasma and serum of 13–15-year-old children.

	Boys						Girls ^a^					
Hormone	*n*	Mean	SEM	Median	Min	Max	*n*	Mean	SEM	Median	Min	Max
E2 (nmol/L plasma)	48 ^b^	0.07	0.01	0.08	0.01	0.15	32	0.37	0.07	0.22	0.09	1.67
LH (U/L plasma)	48 ^c^	3.19	0.21	3.31	0.05	7.45	32	6.98	0.63	7.01	0.14	14.96
FSH (U/L plasma)	48	2.69	0.19	2.51	0.21	5.86	32	4.48	0.39	4.59	1.14	9.57
AMH (ng/mL serum)	54	21.9	3.78	10.86	2.45	111.32	34	3.47	0.34	3.13	0.51	8.51
Inhibin B (pg/mL serum)	54	183.1	7.95	190.52	66.78	328.63	34 ^d^	58.28	6.87	58.86	1.46	190.43
Testosterone (nmol/L plasma)	48	11.37	0.91	12.20	0.27	26.93						
Free Testosterone (nmol/L plasma)	48	0.22	0.02	0.23	0.00	0.58						
SHBG (nmol/L plasma)	48	42.80	3.04	37.60	10.90	106.50						
Albumin (g/L serum)	54	47.49	0.28	47.50	42.70	52.20						

E2: estradiol; LH: luteinizing hormone; FSH: follicle stimulating hormone; AMH: anti-müllerian hormone; SHBG: sex hormone-binding globulin; samples < LOD taken as LOD/2; ^a^ levels of nine girls who uses contraceptives were excluded; ^b^ 9 samples < LOD; ^c^ 1 sample < LOD; ^d^ 2 samples < LOD.

**Table 4:** (**a**) Spearman’s correlation coefficients between pubertal characteristics in boys. (**b**) Spearman’s correlation coefficients between pubertal characteristics in girls.

**(a)**										
	**Tanner Genital Stage**				**Testicular Volume**				**Testosterone**	
	**rho**		***p***		**rho**		***p***		**rho**	***p***
**Tanner Pubic Hair stage**	0.76		<0.001		0.52		<0.001		0.66	<0.001
**Tanner Genital Stage**	x		x		0.51		<0.001		0.49	<0.001
**Testicular Volume**	0.51		<0.001		x		x		0.42	0.004
**(b)**										
	**Tanner Pubic** **Hair Stage**		**Self-Reported Age at Menarche**		**Self-Reported Age at Onset Breast Growth**		**Self-Reported Age at Onset of Pubic Hair Growth**		**Self-Reported Age at Onset Growth Spurt**	
	**rho**	***p***	**rho**	***p***	**rho**	***p***	**rho**	***p***	**rho**	***P***
**Tanner Breast Stage**	0.46	0.003	−0.25	0.154	−0.19	0.241	−0.10	0.537	−0.21	0.232
**Tanner Pubic hair Stage**	x	x	−0.46	0.006	−0.49	0.001	−0.45	0.003	−0.30	0.090

**Table 5:** Univariate linear regression between body mass index (BMI) and reproductive hormones and pubertal characteristics in boys.

	Free Testosterone		E2		LH		SHBG		Testicular Volume		Self-Reported Age at Onset Growth Spurt		Tanner Pubic Hair Stage 5 (Compared to 4)	
	beta	*p*	beta	*p*	beta	*p*	beta	*p*	beta	*p*	beta	*p*	beta	*p*
BMI	0.30	0.037	0.43	0.003	0.36	0.013	−0.51	<0.001	0.24	0.087	−0.32	0.033	0.38	0.006

Only associations with *p* < 0.10 are shown; E2: estradiol; LH: luteinizing hormone; SHBG: sex hormone-binding globulin.

## Data Availability

Data is available on request.

## Supplementary Materials

The following supporting information can be downloaded at: https://www.mdpi.com/article/10.3390/ijerph19159423/s1, Table S1: *T*-tests comparing means of levels of reproductive hormones between the RENCO and the COMPARE cohort for boys included in the DACE study; Table S2: Linear regression analyses for prenatal levels of (hydroxylated) polychlorinated biphenyls (log10 transformed) and reproductive hormone levels in 13–15-year-old boys; Table S3: Linear regression analyses for prenatal levels of (hydroxylated) polychlorinated biphenyls (log10 transformed) and testicular volume and self-reported ages at onset pubertal characteristics in 13–15-year-old boys; Table S4: Linear regression analyses for prenatal levels of (hydroxylated) polychlorinated biphenyls (log10 transformed) and Tanner stages in 13–15-year-old boys; Table S5: Multivariable linear regression analyses for prenatal levels of (hydroxylated) polychlorinated biphenyls (log10 transformed) and reproductive hormone levels in 13–15-year-old girls; Table S6: Linear regression analyses for prenatal levels of (hydroxylated) polychlorinated biphenyls (log10 transformed) and Tanner stages in 13–15-year-old girls; Table S7: Linear regression analyses for prenatal levels of (hydroxylated) polychlorinated biphenyls (log10 transformed) and self-reported ages at onset pubertal characteristics in 13–15-year-old girls; Table S8: Linear regression analyses for prenatal levels of other POPs (log10 transformed) and reproductive hormone levels in 13–15-year-old boys; Table S9: Linear regression analyses for prenatal levels of other POPs (log10 transformed) and testicular volume in 13–15-year-old boys; Table S10: Multivariable linear regression analyses for prenatal levels of other POPs (log10 transformed) and reproductive hormone levels in 13–15-year-old girls.
