# Supplementary material for: Prenatal Environmental Exposure to Persistent Organic Pollutants and Reproductive Hormone Profile and Pubertal Development in Dutch Adolescents
Source: Int J Environ Res Public Health. 2022 Aug 1;19(15):9423. doi: 10.3390/ijerph19159423 (PMC9367960; doi:10.3390/ijerph19159423)
Supplement: Supplementary file 1 [file ijerph-19-09423-s001.zip › ijerph-1777112-supplementary.pdf]

SUPPLEMENTARY TABLES

To: 'Prenatal Environmental Exposure to Persistent Organic Pollutants and Reproductive Hormone Profile and Pubertal Development in Dutch Adolescents'

Authors: S.A. Berghuis, A.F. Bos, H. Groen, W.H.A. de Jong, A.C. Muller Kobold, L. Wagenmakers-Huizinga, P.J.J. Sauer and G. Bocca

Table S1. *T*-tests comparing means of levels of reproductive hormones between the RENCO and the COMPARE cohort for boys included in the DACE study

Hormone	Cohort	*n*	Mean	SD	SEM	t	*P*-value
Testosterone (nmol/L plasma)	RENCO	20	14.06	5.41	1.21	2.64	.01
	COMPARE	28	9.45	6.33	1.20		
Free testosterone (nmol/L plasma)	RENCO	20	0.30	0.12	0.03	4.31	<.001
	COMPARE	28	0.15	0.11	0.02		
E2 [a] (nmol/L plasma)	RENCO	20	0.10	0.04	0.01	4.58	<.001
	COMPARE	28	0.05	0.03	0.01		
LH [b] (U/L plasma)	RENCO	20	3.79	1.44	0.32	2.53	.02
	COMPARE	28	2.76	1.36	0.26		
FSH (U/L plasma)	RENCO	20	3.20	1.30	0.29	2.45	.02
	COMPARE	28	2.32	1.18	0.22		
SHBG (nmol/L plasma)	RENCO	20	32.56	20.86	4.66	-3.10	.00
	COMPARE	28	50.13	18.20	3.44		
Inhibin B (pg/ml serum)	RENCO	26	177.99	59.51	11.67	-0.62	.54
	COMPARE	28	187.85	58.04	10.97		
AMH (ng/ml serum)	RENCO	26	17.06	22.89	4.49	-1.23	.22
	COMPARE	28	26.32	31.47	5.95		
Albumin (g/L serum)	RENCO	26	47.78	1.90	0.37	0.99	.33
	COMPARE	28	47.23	2.16	0.41		

RENCO cohort: Risk of Endocrine Contaminants on Human Health cohort; GIC cohort: Groningen-Infant-COMPARE (Comparison of Exposure-Effect Pathways to Improve the Assessment of Human Health Risks of Complex Environmental Mixtures of Organohalogens) cohort; DACE study: Development at Adolescence and Chemical Exposure study; E2: estradiol; LH: luteinizing hormone; FSH: follicle stimulating hormone; AMH: anti-müllerian hormone; SHBG: sex hormone-binding globulin; samples <LOD taken as LOD/2; [a] 9 samples < LOD; [b] 1 sample <LOD.

Table S2. Linear regression analyses for prenatal levels of (hydroxylated) polychlorinated biphenyls (log10 transformed) and reproductive hormone levels in 13-15-year-old boys

		Testosterone [a] (nmol/L plasma)		Free testosterone [b] (nmol/L plasma)		Estradiol [b,c] (nmol/L plasma)		LH [b,d] (U/L plasma)		FSH [a] (U/L plasma)		SHBG [b] (nmol/L plasma)			AMH [a] (ng/mL serum)		Inhibin B [a] (ng/mL serum)	
**Compound**	***n***	**β**	***P*-value**	**β**	***P*-value**	**β**	***P*-value**	**β**	***P*-value**	**β**	***P*-value**	**β**	***P*-value**	***n***	**β**	***P*-value**	**β**	***P*-value**
**PCB-105 [e]**	20	**0.50**	**0.03**	**0.45**	**0.08**	0.33	0.15	0.06	0.81	-0.15	0.56	-0.12	0.62	26	-0.24	0.29	**0.37**	**0.08**
**PCB-118 [e]**	20	**0.46**	**0.06**	0.40	0.11	0.32	0.16	0.05	0.85	-0.19	0.45	-0.18	0.47	26	-0.30	0.18	**0.45**	**0.03**
**PCB-138 [e]**	20	**0.44**	**0.05**	0.36	0.14	0.33	0.12	-0.10	0.66	-0.23	0.34	-0.12	0.59	26	**-0.36**	**0.08**	0.28	0.18
**PCB-146 [e]**	20	**0.49**	**0.04**	**0.51**	**0.04**	**0.40**	**0.07**	-0.08	0.72	-0.18	0.47	-0.12	0.61	26	-0.24	0.27	0.24	0.27
**PCB-153**	48	0.16	0.28	0.13	0.32	0.05	0.70	0.05	0.74	-0.13	0.40	-0.05	0.70	54	-0.21	0.16	0.06	0.72
**- RENCO**	20	**0.44**	**0.05**	0.39	0.10	0.31	0.14	-0.04	0.86	-0.20	0.39	-0.16	0.49	26	**-0.35**	**0.09**	0.28	0.16
**- GIC**	28	-0.04	0.85	-0.11	0.53	-0.13	0.49	0.01	0.95	-0.16	0.44	0.09	0.64	28	-0.08	0.71	-0.05	0.80
**PCB-156 [e]**	20	**0.61**	**0.00**	**0.53**	**0.02**	**0.37**	**0.07**	0.06	0.78	-0.07	0.76	-0.23	0.29	26	**-0.49**	**0.01**	0.25	0.22
**PCB-170 [e]**	20	**0.55**	**0.01**	**0.47**	**0.04**	**0.37**	**0.07**	-0.03	0.88	-0.02	0.94	-0.17	0.43	26	**-0.43**	**0.03**	0.20	0.33
**PCB-180 [e]**	20	**0.48**	**0.03**	**0.46**	**0.05**	0.34	0.11	0.02	0.95	-0.01	0.98	-0.21	0.36	26	**-0.36**	**0.08**	0.17	0.42
**PCB-183 [e]**	20	0.34	0.15	0.23	0.36	0.20	0.38	-0.04	0.88	**-0.42**	**0.08**	-0.08	0.74	26	-0.24	0.28	**0.44**	**0.03**
**PCB-187 [e]**	20	**0.48**	**0.03**	**0.48**	**0.04**	0.34	0.11	0.03	0.89	-0.16	0.51	-0.18	0.43	26	-0.28	0.18	0.21	0.31
**Σ 10 PCBs [e]**	20	**0.49**	**0.03**	**0.42**	**0.07**	0.34	0.11	-0.04	0.86	-0.18	0.44	-0.17	0.46	26	**-0.38**	**0.06**	0.30	0.14

Table S2 continued

		Testosterone [a] (nmol/L plasma)		Free testosterone [b] (nmol/L plasma)		Estradiol [b,c] (nmol/L plasma)		LH [b,d] (U/L plasma)		FSH [a] (U/L plasma)		SHBG [b] (nmol/L plasma)			AMH [a] (ng/mL serum)		Inhibin B [a] (ng/mL serum)	
**Compound**	***n***	**β**	***P*-value**	**β**	***P*-value**	**β**	***P*-value**	**β**	***P*-value**	**β**	***P*-value**	**β**	***P*-value**	***n***	**β**	***P*-value**	**β**	***P*-value**
**4-OH-PCB-107**	47	-0.01	0.94	-0.03	0.84	0.03	0.83	0.05	0.78	0.07	0.72	-0.02	0.90	53	**-0.31**	**0.07**	-0.13	0.47
**- RENCO**	20	-0.24	0.31	-0.38	0.12	-0.12	0.60	-0.18	0.41	-0.10	0.67	0.32	0.15	26	-0.07	0.75	-0.03	0.88
**- GIC**	27	0.15	0.47	0.18	0.39	0.00	1.00	0.03	0.88	-0.05	0.80	-0.24	0.20	27	**-0.42**	**0.04**	0.03	0.89
**3′-OH-PCB-138** [e]	20	-0.08	0.74	-0.18	0.46	-0.07	0.73	-0.30	0.15	-0.18	0.44	0.16	0.48	26	-0.07	0.74	-0.10	0.62
**4-OH-PCB-146**	48	-0.13	0.33	-0.17	0.18	-0.08	0.49	-0.21	0.14	**-0.28**	**0.05**	0.14	0.27	54	0.00	0.99	0.04	0.80
**- RENCO**	20	-0.22	0.35	-0.25	0.30	-0.06	0.77	-0.32	0.13	-0.26	0.27	0.25	0.27	26	0.00	0.99	0.02	0.91
**- GIC**	28	-0.07	0.74	-0.10	0.65	-0.25	0.22	-0.13	0.55	-0.31	0.13	-0.01	0.98	28	-0.04	0.86	-0.12	0.54
**3-OH-PCB-153** [e]	20	-0.02	0.95	0.00	1.00	-0.06	0.78	-0.03	0.90	-0.22	0.35	0.02	0.94	26	-0.07	0.74	0.05	0.81
**4′-OH-PCB-172** [e]	16	-0.01	0.97	-0.11	0.71	-0.08	0.77	-0.20	0.49	0.38	0.18	0.24	0.36	20	0.04	0.87	-0.13	0.60
**4-OH-PCB-187**	48	-0.18	0.34	-0.14	0.39	0.06	0.71	-0.10	0.59	-0.07	0.72	0.13	0.43	54	0.18	0.32	-0.23	0.22
**- RENCO**	20	-0.22	0.36	-0.21	0.39	0.02	0.92	-0.27	0.23	0.04	0.88	0.22	0.33	26	0.20	0.35	-0.06	0.79
**- GIC**	28	-0.08	0.69	-0.07	0.74	-0.04	0.86	-0.08	0.72	-0.28	0.18	0.01	0.97	28	0.12	0.58	-0.21	0.28
**Σ 6 OH-PCBs** [e]	16	-0.32	0.25	-0.38	0.18	-0.12	0.64	-0.32	0.24	0.06	0.82	0.20	0.43	20	0.03	0.89	-0.13	0.60

LH: luteinizing hormone; FSH: follicle stimulating hormone; SHBG: sex hormone-binding globulin; AMH: anti-müllerian hormone; associations with a *P*-value <.10 are shown in bold; samples <LOD are taken as LOD/2 in analyses; [a] corrected for age at examination; [b] corrected for age and BMI; [c] 9 samples <LOD; [d] 1 samples < LOD; [e] RENCO cohort.

Table S3. Linear regression analyses for prenatal levels of (hydroxylated) polychlorinated biphenyls (log10 transformed) and testicular volume and self-reported ages at onset pubertal characteristics in 13-15-year-old boys

	Testicular volume [a]			Age onset voice change			Age at ejaculation			Onset growth pubic hair			Onset of growth spurt [b]		
**Compound**	*n*	β	***P*-value**	*n*	β	***P* –value**	*n*	β	***P* -value**	*n*	β	***P* -value**	*n*	β	***P* -value**
**PCB-105 [c]**	25	0.24	0.27	20	-0.03	0.91	19	-.25	.30	25	-0.01	0.95	14	**-0.53**	**0.08**
**PCB-118 [c]**	25	**0.39**	**0.06**	20	0.03	0.89	19	-.30	.22	25	-0.01	0.97	14	-0.41	0.19
**PCB-138 [c]**	25	**0.45**	**0.02**	20	-0.36	0.12	19	-.06	.80	25	0.04	0.84	14	**-0.65**	**0.01**
**PCB-146 [c]**	25	**0.44**	**0.03**	20	-0.30	0.19	19	-.24	.32	25	-0.05	0.83	14	**-0.46**	**0.09**
**PCB-153**	54	0.06	0.66	35	-0.06	0.75	29	.12	.54	49	0.18	0.21	31	0.14	0.44
- **RENCO**	25	**0.42**	**0.03**	20	-0.31	0.18	19	-.10	.68	25	0.03	0.88	14	**-0.53**	**0.06**
- **GIC**	29	-0.24	0.21	15	-0.20	0.48	10	-.14	.71	24	0.08	0.71	17	0.18	0.50
**PCB-156 [c]**	25	**0.41**	**0.03**	20	-0.34	0.14	19	-.13	.59	25	-0.01	0.96	14	**-0.53**	**0.06**
**PCB-170 [c]**	25	**0.37**	**0.05**	20	**-0.51**	**0.02**	19	-.04	.86	25	0.09	0.67	14	**-0.66**	**0.02**
**PCB-180 [c]**	25	**0.34**	**0.07**	20	**-0.53**	**0.02**	19	-.14	.58	25	0.01	0.98	14	**-0.55**	**0.04**
**PCB-183 [c]**	25	0.27	0.19	20	-0.30	0.20	**19**	.10	.70	25	0.21	0.32	14	-0.28	0.35
**PCB-187 [c]**	25	**0.33**	**0.09**	20	**-0.48**	**0.03**	19	-.19	.43	25	-0.03	0.90	14	-0.43	0.11
**Σ 10 PCBs [c]**	25	**0.44**	**0.02**	20	-0.35	0.13	19	-.14	.58	25	0.04	0.85	14	**-0.57**	**0.04**

Table S3 continued

	Testicular volume [a]			Age onset voice change			Age at ejaculation			Onset growth pubic hair			Onset of growth spurt [b]		
**Compound**	***n***	**β**	***P*-value**	***n***	**β**	***P* –value**	***n***	**β**	***P* -value**	***n***	**β**	***P* -value**	***n***	**β**	***P* -value**
**4-OH-PCB-107**	53	-0.14	0.39	**34**	**0.30**	**0.09**	**28**	**0.52**	**0.01**	**48**	**0.24**	**0.10**	31	0.22	0.20
- **RENCO**	25	-0.22	0.27	20	0.00	1.00	**19**	**0.60**	**0.01**	25	0.22	0.29	14	-0.19	0.50
- **GIC**	28	0.07	0.72	14	0.45	0.10	9	-0.06	0.87	23	-0.14	0.52	17	0.09	0.75
**3′-OH-PCB-138 [c]**	25	-0.15	0.45	20	-0.31	0.19	19	0.17	0.49	25	-0.07	0.75	14	**-0.55**	**0.04**
**4-OH-PCB-146**	54	-0.19	0.13	**35**	**-0.32**	**0.06**	29	0.13	0.50	49	0.02	0.91	31	-0.07	0.71
- **RENCO**	25	-0.11	0.58	**20**	**-0.47**	**0.05**	19	0.26	0.29	25	0.11	0.61	14	**-0.62**	**0.03**
- **GIC**	29	**-0.37**	**0.06**	15	0.14	0.62	10	-0.07	0.85	24	0.03	0.90	17	0.23	0.38
**3-OH-PCB-153 [c]**	25	-0.17	0.37	20	-0.11	0.63	19	0.19	0.45	25	0.13	0.53	14	-0.09	0.76
**4′-OH-PCB-172 [c]**	20	-0.02	0.93	**15**	**-0.45**	**0.09**	15	0.10	0.74	20	0.03	0.90	10	**-0.68**	**0.09**
**4-OH-PCB-187**	54	-0.24	0.15	35	0.04	0.84	29	0.22	0.25	49	0.20	0.17	31	0.17	0.36
- **RENCO**	25	0.13	0.49	20	-0.36	0.12	19	0.11	0.66	25	0.05	0.83	14	-0.29	0.34
- **GIC**	29	**-0.56**	**0.00**	15	-0.00	1.00	10	-0.37	0.29	24	-0.05	0.83	17	0.06	0.81
**Σ 6 OH-PCBs [c]**	20	-0.15	0.49	15	-0.32	0.25	15	0.25	0.38	20	0.10	0.68	10	-0.66	0.12

Associations with a *P*-value <.10 are shown in bold; only boys who reported 'yes' on the question whether they mentioned onset of pubertal characteristic were included for that specific analyses; [a] corrected for age at examination; [b] corrected for BMI and onset paternal growth spurt; [c] RENCO cohort.

Table S4. Linear regression analyses for prenatal levels of (hydroxylated) polychlorinated biphenyls (log10 transformed) and Tanner stages in 13-15-year-old boys [a]

		**Tanner pubic hair stage [b]**								**Tanner genital stage [c]**							
		**Stage 1**		**Stage 2**		**Stage 3**		**Stage 5**		**Stage 1**		**Stage 2**		**Stage 3**		**Stage 5**	
**Compound**	***n***	**β**	***P* -value**	**β**	***P* -value**	**β**	***P* -value**	**β**	***P* -value**	**β**	***P* -value**	**β**	***P* -value**	**β**	***P* -value**	**β**	***P* -value**
**PCB-105 [d]**	26	x	x	**-0.32**	**0.07**	**-0.50**	**0.01**	0.03	0.86	x	x	-0.12	0.51	-0.28	0.15	**0.33**	**0.07**
**PCB-118 [d]**	26	x	x	**-0.46**	**0.01**	**-0.52**	**0.00**	-0.05	0.79	x	x	-0.18	0.35	-0.27	0.17	**0.31**	**0.10**
**PCB-138 [d]**	26	x	x	**-0.47**	**0.02**	**-0.44**	**0.03**	0.15	0.45	x	x	-0.30	0.14	**-0.40**	**0.06**	0.23	0.23
**PCB-146 [d]**	26	x	x	**-0.51**	**0.00**	**-0.40**	**0.02**	0.18	0.31	x	x	**-0.34**	**0.07**	**-0.45**	**0.02**	0.20	0.24
**PCB-153**	53	0.12	0.39	**-0.29**	**0.04**	-0.16	0.27	0.15	0.28	-0.20	0.16	-0.24	0.17	-0.22	0.17	0.51	0.26
- **RENCO**	26	x	x	**-0.47**	**0.02**	**-0.45**	**0.02**	0.12	0.53	x	x	-0.26	0.22	**-0.37**	**0.09**	0.20	0.31
- **GIC**	27	**0.39**	**0.09**	-0.14	0.56	0.17	0.47	x	x	-0.08	0.78	0.03	0.94	0.23	0.58	x	x
**PCB-156 [d]**	26	x	x	**-0.41**	**0.04**	**-0.44**	**0.02**	0.26	0.19	x	x	-0.25	0.23	**-0.36**	**0.09**	0.26	0.20
**PCB-170 [d]**	26	x	x	**-0.35**	**0.09**	-0.29	0.15	0.33	0.12	x	x	-0.28	0.21	-0.31	0.18	0.17	0.43
**PCB-180 [d]**	26	x	x	**-0.36**	**0.08**	-0.23	0.25	0.26	0.23	x	x	-0.26	0.24	-0.27	0.24	0.11	0.61
**PCB-183 [d]**	26	x	x	-0.04	0.85	**-0.40**	**0.06**	-0.01	0.97	x	x	0.09	0.66	-0.33	0.12	0.18	0.36
**PCB-187 [d]**	26	x	x	**-0.38**	**0.06**	**-0.45**	**0.03**	0.16	0.44	x	x	-0.15	0.48	**-0.43**	**0.05**	0.14	0.50
**Σ 10 PCBs [d]**	26	x	x	**-0.46**	**0.02**	**-0.46**	**0.02**	0.17	0.39	x	x	-0.27	0.19	**-0.39**	**0.07**	0.23	0.23
**4-OH-PCB-107**	52	**-0,25**	**0,05**	0.00	0.99	-0.10	0.44	-0.01	0.94	**-0.37**	**0.00**	-0.13	0.32	-0.14	0.28	**-0.20**	**0.06**
- **RENCO**	26	x	x	0.12	0.61	-0.16	0.49	-0.09	0.70	x	x	-0.10	0.64	-0.05	0.82	**-0.44**	**0.04**
- **GIC**	26	-0.33	0.19	-0.01	0.98	-0.02	0.94	x	x	**-0.47**	**0.06**	0.12	0.72	0.09	0.79	x	x

Associations with a *P*-value <.10 are shown in bold; [a] using Tanner Stage 4 as reference category; [b] corrected for age at examination and BMI; [c] corrected for age at examination; [d] RENCO cohort.

Table S5. Multivariable linear regression analyses for prenatal levels of (hydroxylated) polychlorinated biphenyls (log10 transformed) and reproductive hormone levels in 13-15-year-old girls

		Estradiol [a] (nmol/L plasma)		LH [a,b] (U/L plasma)		FSH [a,b] (U/L plasma)			AMH [a] (ng/mL serum)		Inhibin B [a,c] (ng/mL serum)	
**Compound**	***n***	**β**	***P*-value**	**β**	***P*-value**	**β**	***P*-value**	***n***	**β**	***P*-value**	**β**	***P*-value**
**PCB-105 [d]**	20	0.01	0.98	0.07	0.78	0.21	0.37	22	-0.08	0.73	-0.31	0.14
**PCB-118 [d]**	20	0.02	0.94	0.12	0.36	0.14	0.56	22	0.01	0.98	-0.21	0.31
**PCB-138 [d]**	20	0.12	0.61	0.11	0.64	0.10	0.65	22	0.17	0.47	0.01	0.98
**PCB-146 [d]**	20	0.02	0.92	0.18	0.46	0.29	0.19	22	0.11	0.65	-0.22	0.27
**PCB-153**	32	0.30	0.19	0.01	0.97	-0.16	0.94	34	0.16	0.43	**-0.42**	**0.05**
- **RENCO**	20	0.24	0.30	0.27	0.26	0.42	0.27	22	0.12	0.61	-0.04	0.86
- **GIC**	12	-0.21	0.52	**-0.64**	**0.04**	**-0.62**	**0.03**	12	0.19	0.58	-0.52	0.11
**PCB-156 [d]**	20	0.29	0.22	0.20	0.43	0.22	0.37	22	0.13	0.60	-0.17	0.42
**PCB-170 [d]**	20	0.30	0.17	0.28	0.23	0.31	0.17	22	0.16	0.50	-0.02	0.94
**PCB-180 [d]**	20	**0.44**	**0.06**	0.38	0.14	0.23	0.35	22	0.12	0.65	-0.02	0.95
**PCB-183 [d]**	20	-0.15	0.51	-0.19	0.43	0.25	0.27	22	-0.05	0.83	0.18	0.39
**PCB-187 [d]**	20	0.20	0.39	0.28	0.24	**0.38**	**0.08**	22	0.12	0.61	0.04	0.86
**Σ 10 PCBs [d]**	20	0.22	0.33	0.23	0.33	0.24	0.29	23	0.12	0.62	-0.06	0.76
**4-OH-PCB-107**	31	0.13	0.53	0.00	0.99	-0.31	0.14	33	0.10	0.59	-0.19	0.36
- **RENCO**	19	0.04	0.88	0.14	0.58	-0.24	0.34	21	0.07	0.78	0.03	0.91
- **GIC**	12	-0.36	0.24	-0.19	0.58	0.05	0.88	12	0.11	0.74	0.01	0.97
**3′-OH-PCB-138 [d]**	19	0.17	0.54	-0.14	0.63	-0.07	0.80	21	-0.38	0.16	-0.34	0.16
**4-OH-PCB-146**	31	0.09	0.69	-0.15	0.50	**-0.42**	**0.05**	33	-0.01	0.95	-0.33	0.11
- **RENCO**	19	0.10	0.75	0.01	0.98	-0.29	0.34	21	-0.29	0.39	0.10	0.75
- **GIC**	12	-0.27	0.40	-0.33	0.32	**-0.49**	**0.09**	12	0.26	0.45	-0.42	0.20
**3-OH-PCB-153 [d]**	19	0.27	0.31	0.06	0.82	0.10	0.71	21	-0.38	0.14	-0.13	0.60
**4′-OH-PCB-172 [d]**	15	0.22	0.52	0.05	0.89	-0.16	0.65	16	-0.47	0.15	**-0.46**	**0.06**
**4-OH-PCB-187**	31	0.14	0.49	0.00	0.99	-0.33	0.10	33	0.15	0.43	-0.19	0.33
- **RENCO**	19	0.10	0.72	0.21	0.45	-0.15	0.59	21	0.02	0.95	0.25	0.33
- **GIC**	12	-0.39	0.20	-0.12	0.72	-0.24	0.44	12	0.33	0.32	-0.16	0.65
**Σ 6 OH-PCBs [d]**	15	0.31	0.32	-0.01	0.99	-0.24	0.47	16	-0.41	0.19	-0.37	0.12

LH: luteinizing hormone; FSH: follicle stimulating hormone; AMH: anti-müllerian hormone; associations with a *P*-value <.10 are shown in bold; samples <LOD are taken as LOD/2 in analyses; nine girls used contraceptives and were excluded for these analyses; [a] corrected for age at examination in months; [b] 1 sample <LOD; [c] 3 samples <LOD; [d] RENCO cohort.

Table S6. Linear regression analyses for prenatal levels of (hydroxylated) polychlorinated biphenyls (log10 transformed) and Tanner stages in 13-15-year-old girls [a]

	Tanner pubic hair stage [b]					Tanner breast stage [c]				
	n	Stage 3		Stage 5		*n*	Stage 3		Stage 5	
**Compound**		β	*P* -value	β	*P* -value		β	*P* -value	β	*P* -value
**PCB-105 [d]**	24	-0.27	0.31	-0.19	0.41	27	0.03	0.86	**0.47**	**0.02**
**PCB-118 [d]**	24	**-0.42**	**0.08**	-0.07	0.72	27	-0.18	0.31	**0.53**	**0.01**
**PCB-138 [d]**	24	-0.31	0.18	-0.08	0.67	27	**-0.53**	**0.01**	0.19	0.29
**PCB-146 [d]**	24	-0.24	0.36	0.14	0.45	27	-0.24	0.21	**0.36**	**0.07**
**PCB-153**	39	**-0.55**	**0.00**	-0.07	0.65	42	-0.16	0.27	**0.31**	**0.03**
- **RENCO**	24	**-0.44**	**0.07**	-0.23	0.26	27	**-0.48**	**0.01**	**0.32**	**0.07**
- **GIC**	15	**-0.45**	**0.09**	x	x	15	0.18	0.55	x	x
**PCB-156 [d]**	23	**-0.53**	**0.03**	-0.28	0.19	26	-0.23	0.18	**0.37**	**0.03**
**PCB-170 [d]**	23	**-0.49**	**0.04**	**-0.41**	**0.06**	26	**-0.37**	**0.07**	0.25	0.20
**PCB-180 [d]**	23	-0.36	0.13	**-0.43**	**0.05**	26	**-0.38**	**0.07**	0.21	0.29
**PCB-183 [d]**	24	0.15	0.58	-0.08	0.73	27	0.15	0.51	0.02	0.94
**PCB-187 [d]**	24	-0.33	0.21	-0.16	0.48	27	-0.27	0.20	0.17	0.43
**Σ 10 PCBs [d]**	23	**-0.41**	**0.10**	-0.24	0.26	26	**-0.41**	**0.03**	**0.35**	**0.06**
**4-OH-PCB-107**	36	-0.09	0.64	0.20	0.27	39	0.10	0.57	0.16	0.37
- **RENCO**	22	0.12	0.68	0.16	0.52	25	-0.04	0.87	0.11	0.63
- **GIC**	14	0.06	0.86	0.08	0.80	14	**0.57**	**0.09**	x	x
**3′-OH-PCB-138 [d]**	22	0.04	0.10	0.21	0.36	25	-0.24	0.28	-0.07	0.76
**4-OH-PCB-146**	37	0.24	0.21	0.09	0.63	40	0.18	0.31	-0.03	0.85
- **RENCO**	22	**0.46**	**0.09**	0.24	0.30	25	-0.14	0.40	-0.19	0.25
- **GIC**	15	-0.04	0.88	x	x	15	**0.56**	**0.06**	x	x
**3-OH-PCB-153 [d]**	22	0.35	0.22	0.02	0.93	25	-0.26	0.27	-0.09	0.69
**4′-OH-PCB-172 [d]**	16	0.41	0.18	0.08	0.77	19	-0.35	0.15	-0.20	0.41
**4-OH-PCB-187**	37	-0.02	0.93	0.14	0.45	40	0.17	0.36	-0.04	0.81
- **RENCO**	22	0.24	0.40	0.07	0.77	25	-0.05	0.77	-0.23	0.18
- **GIC**	15	0.06	0.83	x	x	15	**0.65**	**0.03**	x	x
**Σ 6 OH-PCBs [d]**	16	0.27	0.39	0.10	0.74	19	**-0.46**	**0.04**	-0.10	0.66
**PCP**	15	-0.32	0.24	x	x	15	**-0.58**	**0.06**	x	x
**BDE-154**	14	**-0.61**	**0.02**	x	x	14	-0.18	0.59	x	x

Associations with a *P*-value <.10 are shown in bold; [a] using Tanner Stage 4 as reference category; [b] corrected for maternal age at maternal menarche; [c] corrected for age at examination; [d] RENCO cohort; BDEs 47, 99, 100, 153, DDE and HBCDD were not significantly associated with Tanner stages in girls.

Table S7. Linear regression analyses for prenatal levels of POPs (log10 transformed) and self-reported ages at onset pubertal characteristics in 13-15-year-old girls

	Onset breast growth [a]			Onset growth spurt			Onset growth pubic hair			Age at menarche [a]		
**Compound**	***n***	**β**	***P*-value**	***n***	**β**	***P* –value**	***n***	**β**	***P* -value**	***n***	**β**	***P* -value**
**3′-OH-PCB-138 [e]**			ns	22	**-0.37**	**0.09**			ns			ns
**4′-OH-PCB-172 [e]**	18	**0.36**	**0.09**			ns			ns			ns
**4-OH-PCB-187**			ns			ns			ns			ns
- **RENCO**			ns	22	**-0.43**	**0.05**			ns			ns
- **GIC**			ns			ns			ns	12	**0.53**	**0.07**
**BDE-99**	16	**0.43**	**0.09**			ns			ns			ns
**BDE-153**			ns			ns	16	**0.47**	**0.07**			ns
**HBCDD**			ns	12	**-0.53**	**0.07**			ns			ns

Only associations with a *P*-value <.10 are shown; ns = not significant; [a] only children who reported 'yes' on the question whether they mentioned onset of the pubertal characteristics were included; [a] corrected for maternal age at maternal menarche; [e] RENCO cohort; all 10 PCBs, Σ 10 PCBs, OH-PCBs 107, 146, 153, Σ 6 OH-PCBs, BDEs 100 and 154, DDE and PCP were not significantly associated with self-reported ages at onset pubertal characteristics in girls.

Table S8. Linear regression analyses for prenatal levels of other POPs (log10 transformed) and reproductive hormone levels in 13-15-year-old boys

	**Testosterone [a] (nmol/L plasma)**			**Free testosterone [b] (nmol/L plasma)**		**Estradiol [b.c] (nmol/L plasma)**		**LH [b.d] (U/L plasma)**		**FSH [a] (U/L plasma)**		**SHBG [b] (nmol/L plasma)**		**AMH [a] (ng/mL serum)**			**Inhibin B [a] (ng/mL serum)**	
**Compound**	***n***	**β**	***P*-value**	**β**	***P*-value**	**β**	***P*-value**	**β**	***P*-value**	**β**	***P*-value**	**β**	***P*-value**	***n***	**β**	***P*-value**	**β**	***P*-value**
**p,p′-DDE**	28	0.00	1.00	0.01	0.97	-0.11	0.60	-0.06	0.78	-0.26	0.18	**-0.46**	**0.02**	28	-0.16	0.44	-0.02	0.92
**PCP**	28	0.02	0.94	0.01	0.98	0.02	0.93	-0.01	0.98	0.23	0.24	0.07	0.71	28	0.04	0.85	**-0.42**	**0.02**
**BDE-47**	17	0.03	0.93	0.01	0.96	0.13	0.66	-0.06	0.83	-0.07	0.80	-0.39	0.10	18	-0.09	0.73	0.03	0.93
**BDE-99**	15	-0.04	0.89	-0.09	0.77	0.03	0.90	-0.19	0.52	0.17	0.56	-0.11	0.67	17	0.32	0.29	-0.24	0.41
**BDE-100**	16	0.19	0.51	0.06	0.84	0.18	0.50	-0.09	0.73	0.15	0.58	-0.28	0.23	18	-0.18	0.53	-0.09	0.76
**BDE-153**	18	-0.11	0.67	-0.19	0.47	-0.16	0.54	-0.36	0.15	**-0.48**	**0.05**	-0.18	0.43	18	-0.10	0.70	0.22	0.38
**BDE-154**	18	0.34	0.18	**0.44**	**0.08**	0.35	0.16	0.38	0.13	0.11	0.67	**-0.52**	**0.01**	18	-0.38	0.13	-0.05	0.85
**HBCDD**	18	**-0.42**	**0.10**	-0.42	0.10	-0.25	0.34	-0.33	0.19	-0.42	0.10	0.06	0.78	18	0.28	0.29	-0.32	0.21

LH: luteinizing hormone; FSH: follicle stimulating hormone; SHBG: sex hormone-binding globulin; AMH: anti-müllerian hormone; associations with a *P*-value <.10 are shown in bold; samples <LOD are taken as LOD/2 in analyses; [a] corrected for age at examination; [b] corrected for age and BMI; [c] 8 samples <LOD; [d] 1 sample < LOD.

Table S9. Linear regression analyses for prenatal levels of other POPs (log10 transformed) and testicular volume in 13-15-year-old boys

	Testicular volume [a]			Age onset voice change			Age at ejaculation			Onset growth pubic hair			Growth spurt [b]		
**Compound**	***n***	**β**	***P*-value**	***n***	**β**	***P* -value**	***n***	**β**	***P* -value**	***n***	**β**	***P* -value**	***n***	**β**	***P* -value**
**p,p′-DDE**	29	**-0.31**	**0.10**	15	0.22	0.44	10	-0.18	0.61	24	0.06	0.79	17	-0.04	0.89
**PCP**	29	**-0.37**	**0.05**	15	-0.15	0.60	10	-0.45	0.19	24	-0.30	0.16	17	0.19	0.47
**BDE-47**	18	-0.28	0.28	9	-0.06	0.88	6	0.43	0.40	15	0.08	0.78	11	0.15	0.67
**BDE-99**	16	-0.15	0.61	8	-0.59	0.13	5	-0.06	0.92	14	-0.21	0.47	11	0.11	0.75
**BDE-100**	17	-0.07	0.80	8	**-0.63**	**0.09**	5	-0.09	0.88	14	-0.27	0.36	11	0.14	0.71
**BDE-153**	19	0.01	0.97	9	-0.07	0.85	6	0.12	0.83	15	0.08	0.77	11	0.34	0.33
**BDE-154**	19	0.10	0.68	9	0.38	0.32	6	0.17	0.74	15	-0.20	0.48	11	-0.54	0.10
**HBCDD**	19	0.14	0.59	9	0.03	0.93	6	0.49	0.33	15	0.21	0.46	11	0.15	0.69

Associations with a *P*-value <.10 are shown in bold; [a] Only children who reported 'yes' on the question whether they mentioned onset of the pubertal characteristics were included; [a] corrected for age at examination; [b] corrected for BMI and onset paternal growth spurt.

Table S10. Multivariable linear regression analyses for prenatal levels of other POPs (log10 transformed) and reproductive hormone levels in 13-15-year-old girls

	Estradiol [a] (nmol/L plasma)			LH [a] (U/L plasma)		FSH [a] (U/L plasma)		AMH [a] (ng/mL serum)		Inhibin B [a] (ng/mL serum)	
**Compound**	***n***	**β**	***P* -value**	**β**	***P* -value**	**β**	***P* -value**	**β**	***P* -value**	**β**	***P* -value**
**p,p′-DDE**	12	-0.44	0.15	-0.34	0.32	-0.26	0.41	0.35	0.30	-0.17	0.61
**PCP**	12	**0.71**	**0.01**	0.20	0.57	0.21	0.52	-0.46	0.17	0.32	0.36
**BDE-47**	10	-0.02	0.95	0.39	0.31	-0.04	0.91	-0.01	0.97	-0.04	0.92
**BDE-99**	10	0.04	0.91	0.49	0.19	0.41	0.24	0.19	0.63	0.53	0.15
**BDE-100**	10	-0.55	0.10	0.11	0.78	0.10	0.78	0.20	0.59	0.04	0.93
**BDE-153**	10	0.24	0.54	-0.08	0.86	-0.08	0.84	0.02	0.96	0.09	0.83
**BDE-154**	10	-0.09	0.81	-0.13	0.74	-0.28	0.44	-0.13	0.73	-0.42	0.27
**HBCDD**	10	0.29	0.43	0.34	0.39	0.38	0.28	0.08	0.83	**0.72**	**0.04**

LH: luteinizing hormone; FSH: follicle stimulating hormone; AMH: anti-müllerian hormone; associations with a *P*-value <.10 are shown in bold; [a] corrected for age at examination in months.
